# *In vivo* production of entomopathogenic nematodes using *Galleria mellonella*: costs and effect of diets on nematode pathogenicity

**DOI:** 10.21307/jofnem-2019-066

**Published:** 2019-10-05

**Authors:** Régina Kotchofa, Hugues Baimey

**Affiliations:** 1 Department of Crop Production, University of Parakou, Benin

**Keywords:** Diet, Entomopathogenic nematode, *Galleria mellonella*, Mass-rearing, Production cost

## Abstract

Five separate diets – beeswax (BW), glycerol (Gly), and three types of dog croquettes (DC1, DC2, and DC3) – were used to rear larvae of the greater wax moth, *Galleria mellonella.* The larvae were later inoculated with five different isolates of entomopathogenic nematodes (EPN) emerging from the insect larvae reared on the five diets. Insect cadavers were then conserved for 1, 2, 3, and 4 wks (T1, T2, T3, and T4, respectively) to evaluate the nematodes’ progeny production and to assess the pathogenicity of emerging nematodes to *G. mellonella* larvae. Larvae fed on DC1 weighed the least (0.18 ± 0.03 g) and those fed on Gly weighed the most (0.22 ± 0.04 g). Gly was effective for insect development but was the most expensive to produce at 6.30 US dollars/kg. No significant difference (*P* = 0.851) was observed between mortality rates of larvae for nematode isolates for the three best diets (Gly, BW, and DC1) during 1 wk (T1) and 3 wks (T3) after processing at 3 d post-inoculation. All nematode isolates emerged and had the highest population density per insect larva at T1 for isolate Ze4 (*Heterorhabditis sonorensis*) on BW (553.63 ± 311.97 infective juveniles (IJs)/50 μl of suspension, 276,815 IJs/larva) and at T2 on DC1 (488.63 ± 321.37 IJs/50 μl, 244,315 IJs/larva) and for isolate Aglali (*H. sonorensis*) at T1 on Gly (615.18  ±  309.63 IJs/50 μl, 307,590 IJs/larva). This study shows the costs and effectiveness of different diets on development and production of *G. mellonella* larvae and the EPN produced *in vivo*.

The use of biological agents, such as entomopathogenic nematodes (EPN) of the genera *Heterorhabditis* and *Steinernema*, is one of a range of tools for biological control. These nematode species are obligate parasites, lethal, and easy to apply (San-Blas, 2013). They can be mass-produced using *in vivo* or *in vitro* (solid or liquid) culture methods (Gaugler and Han, 2002; Rahoo et al., 2019), but *in vivo* is the method of choice for laboratory-scale production (e.g. for generating material for field trials) according to Shapiro-Ilan et al. (2002). *In vivo* nematode production yields nematodes with good virulence potential (Shapiro-Ilan et al., 2000). Under field conditions, application of EPN in insect host cadavers can reduce the quantity of nematodes required for control per unit area compared with their application in water or other solvents (Shapiro-Ilan et al., 2012). The typical host used to mass-produced EPN is the greater wax moth *Galleria mellonella* L. (Lepidoptera: Pyralidae) (Testa and Shields, 2017).

*Galleria mellonella* is a pest of bee hives and stored beeswax (Nurullahoglu and Susurluk, 2001; Chandel et al., 2003; Jorjão et al., 2018) and has been the subject of several studies, including a search for adequate techniques for its production. The advantages of using *G. mellonella* larvae in nematode-related studies include high susceptibility of the larvae to EPN (Fuchs et al., 2010; Ramarao et al., 2012), their size and short lifecycle, easy rearing on artificial diets consisting of several ingredients, rearing at various temperatures (20-37°C), and high nematode yields (Van Zyl and Malan, 2015; Testa and Shields, 2017; Pereira et al., 2018; Rahoo et al., 2018). The insect pupae and adults do not require feeding (Jorjão et al., 2018). Rearing the insects in darkness can increase mating and reproduction (Jorjão et al., 2018) because they are active at night (Ellis et al., 2013; Kwadha et al., 2017).

The *in vivo* culturing process is costly (Divya and Sankar, 2009), and it is imperative to find alternative solutions to economically rear the insects. Few experimental diets have been entirely successful in replacing their natural diet (Cohen, 2004). In addition to an assessment of the cost, it is important to know whether the diet influences the effectiveness of EPN emerging from an infected diet-reared host to kill and multiply in the target pest species. According to Zhen et al. (2018), the quality of the insect host can affect the efficacy or persistence of EPN produced *in vivo*. Ramakuwela et al. (2014) indicated that insect nutrition has a link to EPN production.

Many studies have tried to optimize the mass-rearing of *G. mellonella*, taking into account the cost and availability of diet ingredients, as well as the ability of the insect to adapt to diets without seriously affecting its development (Coskun et al., 2006; Birah et al., 2008; Kulkarni et al., 2012). The proportion and/or selection of ingredients in the diets play an important role in the development of larvae, as well as in the fitness and quality of the nematodes obtained from them (Shapiro-Ilan et al., 2008). Shapiro-Ilan et al. (2004) also reported that *in vivo* production yields vary greatly among different insect hosts and nematode species. For example, to rear *G. mellonella*, Brighenti et al. (2005) used 250 g of corn meal, 150 g of yeast extract, 100 g of soy ﬂour, 100 g of powder milk, 200 g of honey, 200 g of glycerol, and beeswax blocks. Van Zyl and Malan (2015) tested and compared several other diets based on cereals, beeswax, or chemical compounds. They found that diet 1 (composed of 118 g wheat flour, 206 g wheat bran, 118 g milk powder, 88 g yeast, 24 g wax powder, 175 ml honey, and 175 ml glycerol) produced the heaviest larvae (0.19 g/ larva); and diet 3 (190 ml glycerol, 190 g yeast, 570 g wheat bran) produced the lightest larvae (0.08 g/larva). In the laboratory, a glycerol-based diet is generally used for *G. mellonella* rearing and results in good larval production. Unfortunately, several of the ingredients that constitute the diet such as glycerol, powdered milk, and yeast are expensive and are beyond the means of low-income sweet potato producers who are the main users of EPNs as biological control agents. The costs associated with rearing insects tend to make *in vivo* culture the least cost-efficient approach, compared with approaches such as *in vitro*-solid and *in vitro*-liquid culture (Shapiro-Ilan et al., 2004; Shapiro-Ilan et al., 2012). These considerations have created the need to find strategies to reduce the costs of rearing insects.

Commercially available dog croquettes are used to rear *G. mellonella* and are often composed of different ingredients of various origins (Fuchs et al., 2018). For example, Ellis et al. (2013) described *G. mellonella* breeding using a diet derived by mixing the following ingredients: seven parts (by volume) dry dog croquettes, one part water, and two parts honey, followed by content adjustment with vitamin A to produce whitish larvae. Van Zyl and Malan (2015) used dog croquettes to rear the insects by mixing 345 g of dry dog diet, 85 g of rolled oats, 85 g of wheat bran, 35 g of brewer’s yeast, 190.5 ml of honey, and 165 ml of glycerol.

This study was initiated to find an alternative artificial, environmentally safe, cost effective, and efficient diet for rearing *G. mellonella* larvae and to evaluate the effect of the diets on EPN virulence and multiplication. Specifically, the study (i) determined which among five different diets was best for rearing *G. mellonella*, (ii) evaluated costs of production of the diets, and (iii) assessed under laboratory conditions the virulence of five EPN isolates multiplied on *G. mellonella* larvae fed on the three best diets.

## Materials and methods

### Mass-rearing of G. mellonella and EPN multiplication

#### Diets

Five diets were used in this study: beeswax (BW), standard glycerol-based artificial diet (Gly), and three varieties of adult dog croquettes (DC1-DC3). Compositions of the diets are presented in Table 1.

**Table 1.** Composition of diets and quantity of ingredients.

Diet	Diet code	Ingredients composing the diet	Quantity of ingredients (%)
Beeswax	BW	Beeswax slightly air-dried	100
Standard diet	Gly	Corn flour	18.85
		Wheat flour	18.85
		Soybean flour	9.42
		Powdered milk	14.28
		Yeast	4.76
		Beeswax	15.00
		Honey	9.42
		Glycerol	9.42
Dog croquettes 1	DC1	Beef	4 in the nuggets with beef
		Chicken	4 in the nuggets with chicken
		Carrots	4 in the nuggets with carrots
		Green vegetables	4 in the nuggets with green vegetables
		Derivatives of vegetables, oils and fats, minerals	Not available
Dog croquettes 2	DC2	Cereals	4 in the nuggets with cereals in the form of beige pastille
		Beef dehydrated	4 in the nuggets with beef in the form of red steak
		Poultry dehydrated	4 in the nuggets with chicken in the form of beige steak
		Derivatives of vegetable origin, meats, oils and fats, minerals	Not available
Dog croquettes 3	DC3	Cereals	40
		Meats and derivatives of animal origin	4 of dehydrated beef protein
		Derivatives of vegetable origin	4 of peas
		Oils and fats, minerals, propylene glycol, sugar	Not available

The information on DC1, DC2, and DC3 ingredients and their respective quantities (columns 3 and 4) was taken from the packages containing the croquettes.

The BW was obtained from an apicultural site at Komiguea village in the district of Parakou, Benin. The moisture of BW was measured in the laboratory using a HTC-1 H596 LCD digital temperature and humidity meter (Zhangzhou KASUN Electronic Technology Co. Ltd, China). When the BW was too wet, it was hand-pressed to remove honey and then air-dried for 1 to 3 d in sunlight for 5 to 7 hr per day according to its moisture content. Moisture content was reduced to below 30% to hinder any development of fungi inside the breeding boxes. The insect larvae were directly reared on this natural diet according to Nurullahoglu and Susurluk (2001) and Jorjão et al. (2018).

The Gly diet was used in the laboratory for mass-rearing *G. mellonella* according to Shaik et al. (2017). Glycerol was used as a humectant due to its hygroscopic characteristics and also for its role as a preservative in diets (Pagliaro and Rossi, 2008). Corn and soybean were ground to obtain a flour consistency. All ingredients except honey and glycerol were weighed individually and mixed in a clean, dry aluminum container. For 1 kg of diet, 94.2 g each of glycerol and honey were used; due to their high viscosity, they were then melted in turn and mixed with other ingredients (Table 1). All ingredients were well homogenized by hand (Shaik et al., 2017). The mixture was cooled in ambient air for 1 hr and the diet introduced in breeding boxes for insect breeding. Any surplus mixture was kept in 5-l plastic boxes at 15°C.

Croquettes DC1, DC2, and DC3 were branded ‘Croquettes Adulte (Bœuf, Poulet, Légumes)’, ‘Multicroquettes Chien Adulte (au bœuf, à la volaille, aux céréales)’, and ‘Tendres croquettes Adultes (Au bœuf, aux céréales et aux légumes)’, respectively, with corresponding distributors ‘Belle France’, ‘Bien Vu!’, and ‘U’. Prior to use, the three types of dog croquettes were blended separately in a kitchen blender, mixed with 300 ml of honey per 1 kg of croquettes, and transferred into the breeding boxes for the insects to feed on. Honey was added to the diets to avoid dehydration.

#### Mass-rearing of *G. mellonella*

Larvae of *G. mellonella* were initially reared at ambient temperature (26.66–30.06°C) in 25-l plastic breeding boxes using the Gly diet. To prepare the cylindrical breeding boxes, a 6-cm-diameter hole was drilled in the circular surface of the boxes and used for the transfer of adult *G. mellonella*. The hole was covered with adhesive paper to prevent insects escaping. The lids of the breeding boxes were perforated and their internal surface was covered with fine mesh lined with white tissue to provide aeration.

The Gly diet was placed in an aluminum container, which was introduced into the breeding boxes. Then crumbled BW was placed onto the diet. In total, 15 rectangular (12 cm long and 5 cm wide) pieces of folded white paper were suspended on the white tissue that lined the lids, on the inner wall of breeding boxes at the rate of five pieces per place, using strong glue. Five pieces of folded paper were also placed directly on the diet to serve as a nesting support for insect females (Ramarao et al., 2012). Adult insects were introduced into the breeding boxes, which were then covered with black cloth and maintained in complete darkness (Mohamed et al., 2014) to promote activity in these essentially nocturnal moths. On average, the duration of the life cycle from egg to adult varies from weeks to months (Kwadha et al., 2017). Adults were collected from the breeding boxes to carry out trials with different diets.

#### Cost of producing *G. mellonella* diets

The cost of producing each diet tested was evaluated (Table 2). For Gly, the quantities of ingredients were first determined per kilogram of diet. Then, the cost of purchasing the different ingredients separately was related to the amount of ingredients needed to prepare 1 kg of diet. The total cost of production of the diet was finally estimated by adding the purchase cost of the various ingredients. The BW was supplied per kilogram upon purchase, which means that this represented the direct cost because no other ingredient was added. The dog croquettes were purchased in bags of 4 kg at supermarkets and the cost per kilogram was used in calculating the total cost of the diet with the added cost of honey required. The relationship between costs of producing *G. mellonella* diets and nematode population densities was determined.

**Table 2.** Cost of production of diets tested to rear *G. mellonella* larvae.

Diet code	Composition	Purchase price (USD)^a^	Ingredients to produce 1 kg of diet	Calculated cost of diet/kg (USD)	Cost of total diet/kg (USD)	Time (wks) to feed 20 larvae of *G. mellonella*/kg of diet
BW	Beeswax	5.11/kg	1 kg	5.11	5.11	3
Gly	Corn flour	0.34/kg	188.57 g	0.068		
	Wheat flour	17.03/bag of 25 kg	188.57 g	0.14		
	Soy flour	0.51/kg	94.28 g	0.051		
	Powdered milk	45.97/cardboard box of 10 kg	142.86 g	0.66	6.30	1½
	Yeast	40.86/cardboard box of 10 kg	47.24 g	0.20		
	Beeswax	5.11/kg	150 g	0.77		
	Honey	4.26/liter	94.28 g	0.40		
	Glycerol	42.57/liter	94.28 g	4.02		
DC1	Adult dog multi-rolls + honey	6.81/bag of 4 kg dog croquettes	700 g	1.19		4
		5.11 for 1 liter of honey	300 ml	1.53	2.72	
DC2	Adult dog croquettes + honey	7.66/bag of 4 kg	700 g	1.35	2.88	4
		5.11 for 1 liter of honey	300 ml	1.53		
DC3	Tender adult dog croquettes + honey	8.43/bag of 4 kg	700 g	1.48	2.11	-
		5.11 for 1 liter of honey	300 ml	1.53		

^a^United States dollar; costs based on 2018 retail prices.

#### EPN multiplication

*Galleria mellonella* larvae fed with different diets were inoculated with five different nematode isolates. The effects of the different diets on virulence (mortality to the host and reproduction) of nematodes that emerged from cadavers of *G. mellonella* larvae were assessed.

The EPN isolates belonging to genera *Heterorhabditis* and *Steinernema* including three indigenous isolates (Aglali, Ze4, and Bembereke) and two isolates (*S. carpocapsae* and *S. riobrave*) imported from eNema, Germany were used in this study. The indigenous nematodes (Aglali and Ze4 both *H. sonorensis*; and Bembereke, an unidentified species of genus *Steinernema*) were previously extracted from soil samples collected in the southern and central parts of Benin during diagnostic surveys (Zadji et al., 2013). The imported nematode isolates *S. carpocapsae* and *S. riobrave*, known as pathogens of *G. mellonella* (Shapiro-Ilan et al., 2005; Christen et al., 2007), were included in the study for data comparison. For this study, all nematode isolates were multiplied *in vivo* using *G. mellonella* larvae (Kaya and Stock, 1997; Van Zyl, 2012). After inoculation with nematodes, *G. mellonella* cadavers were transferred into White traps (White, 1927) at 72 hr post-inoculation. Emerging infective juveniles (IJs) were harvested 10 d later and conserved at 15°C for use in experiments (Zadji et al., 2014).

### Virulence of EPN emerged from infected G. mellonella larvae fed on diets

The three diets (BW, Gly, and DC1) that produced the heaviest and greatest numbers of *G. mellonella* larvae identified in the previous assay in the breeding experiment were used for the virulence assay. Eppendorf tubes (2 ml) were used for the EPN virulence assay (Zadji et al., 2014). Each tube was perforated to allow air exchange and was filled to 3/4 of its capacity with previously sterilized sandy soil (85°C, 72 hr) and adjusted to 10% moisture (w/w) (Zadji et al., 2014). We use the five nematode isolates that emerged from infected *G. mellonella* and kept at 15°C as described earlier in the EPN multiplication methodology section. Before their use, IJs were acclimatized to room temperature (28 ± 2°C) for 1 hr and their viability (movement) was checked under a stereomicroscope (20×). Under the same device, 20 IJs in 200-µl water suspensions were transferred into each Eppendorf tube with a Microlux pipette (10-100 µl). A late-stage larva of *G. mellonella* taken from each type of diet was individually introduced into the Eppendorf tube, which was then closed. There were 10 replicates and each comprised one Eppendorf tube per nematode isolate and per diet tested. The tubes were arranged in a completely randomized design and stored at room temperature of 28 ± 2°C in darkness as described in the study of Dolinski et al. (2006). Three days later, dead and living larvae were removed from Eppendorf tubes and counted. Then, larvae were rinsed with tap water to remove nematodes from their surfaces and transferred into 9-cm petri dishes lined with tissue paper. The larvae of the same treatment in a dish did not touch each other. They were kept in the dishes for four exposure times: 1, 2, 3, or 4 wks (T1, T2, T3, and T4, respectively). After each exposure time, half of the population of dead insects per treatment was individually dissected in a 9-cm petri dish under a stereomicroscope (20×). All nematodes inside each insect larva were collected in a 100-ml beaker by rinsing the petri dish to ensure no nematodes remained. The volume of nematode suspensions was adjusted to 25 ml by adding distilled water (Glazer and Lewis, 2000) and homogenized. For each *G. mellonella* larva, three sub-samples, each of 50-μl aliquot of nematodes, were withdrawn with a Microlux pipette (10-100 µl) and placed into three different new 9-cm petri dishes. To count the number of nematodes in petri dishes, 10 ml of distilled water was added to each dish. The second half of the population of dead insects per treatment was transferred into White traps in 9-cm-diameter sterilized petri dishes (1 insect/dish) and progeny production assessed 1 wk later. The suspensions were collected in a 100-ml beaker and the volume of nematode suspensions was adjusted to 25 ml and homogenized. Nematodes were counted from three 50-µl aliquots as described previously for dissection. The nematode population densities presented in this study are the average of the three 50-μl counts of nematodes by dissection or by White traps. The experiment was repeated once using new batches of the same nematode isolates and of *G. mellonella* larvae.

### Effect of diets on G. mellonella development and reproduction

The five diets described above (BW, Gly, and DC1-DC3) were used to determine the most favorable diet for the development (in terms of larvae weight) and reproduction (in terms of progeny production) of *G. mellonella*. For each diet, 2 kg was taken and introduced into 25-l breeding boxes. In total, three breeding boxes were used for each diet. In total, 30 adult insects (25 females and 5 males) (Baimey et al., 2017) were introduced into each breeding box.

Emergence of larvae began 5 d after transfer of adults to the breeding boxes and continued daily according to diets and lasted a further 20 d. Daily monitoring was carried out to check for emergence of young larvae and larvae spinning cocoons. From the appearance of the first cocoon in the breeding boxes, the number of larvae was counted daily until there was no larva without a cocoon. For all diets tested, several larvae reached the cocooning stage almost at the same time before the first adults started to emerge. Thus, in a batch of a minimum of 50 cocoons per diet, 30 larvae were randomly collected the same day from cocoons produced from each diet and their spinning was removed. The larvae were weighed individually and the mean weight of larvae recorded. These larvae were then reintroduced into their respective breeding boxes where they formed new cocoons. The test was repeated again with another batch of larvae.

### Statistical analysis

The mean mortality of insect larvae was tested for normality and homogeneity of treatment variances using Levene’s test. Mortality rates (percentage) of insects due to EPN isolates were corrected according to Abbott (1925). To stabilize the variance of means, mortality data (%) were transformed with square-root arc sine (arc sin √*x*) (Gomez, 1984) prior to analysis of variance (ANOVA). Nematode population density was log_10_(*x* + 1) transformed to normalize the data prior to analyses. Data were subjected to ANOVA using R (version 3.5.1). The differences between treatment means were compared at *P* < 0.05 using the Student–Newman–Keuls test.

A linear bootstrapping regression was performed using 1,000 replicates to test the relationship between costs of diets and nematode population density. The bootstrap package Leisch 2019 in R 3.5.2 (R Core Team 2018) was used.

## Results

### *Galleria mellonella* larvae development on each diet

**Figure 1:**
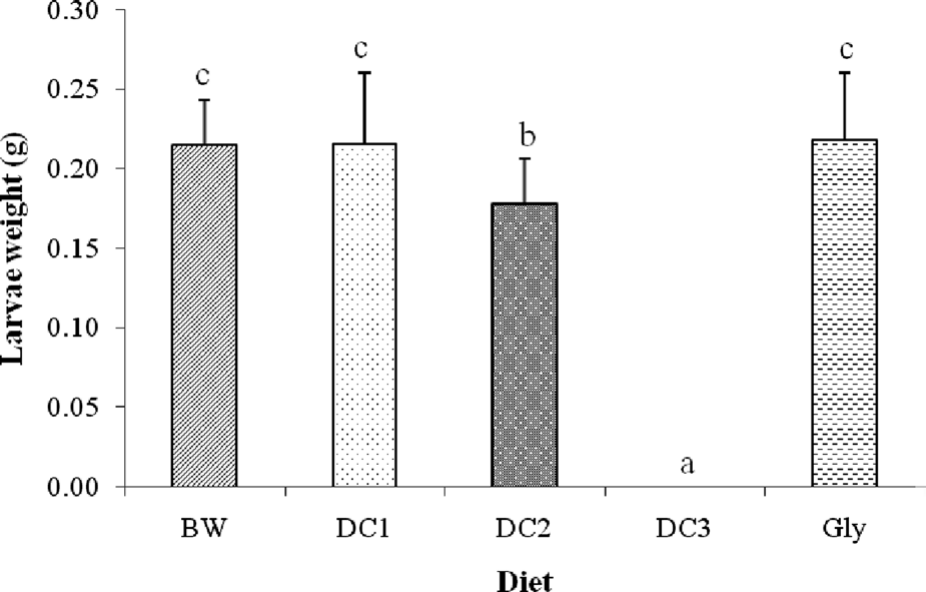
Weight (mean ± standard deviation) of *G. mellonella* larvae obtained at the cocoon spinning stage by diet tested. No larvae emerged on diet DC3, and so there were no data. Weights followed by the same letter do not significantly differ at *P* < 0.05. BW, beeswax; Gly, standard glycerol-based artificial diet; DC1 to DC3, three varieties of adult dog croquettes.

Figure 1 shows the mean weight of *G. mellonella* larvae per diet at the cocooning stage. There were significant differences (*F* = 242.5, df = 4, *P* < 2e−16) between the weights of the larvae obtained per diet. No larvae were observed in DC3. Larvae fed on DC2 had the lowest weight (0.18 ± 0.03 g) (mean ± SD) and larvae fed on BW, DC1, and Gly showed similar (*P* < 0.05) higher weights (0.21 ± 0.03, 0.21 ± 0.04, and 0.22 ± 0.04 g, respectively).

### Influence of diet on *G. mellonella* larvae rearing

**Figure 2:**
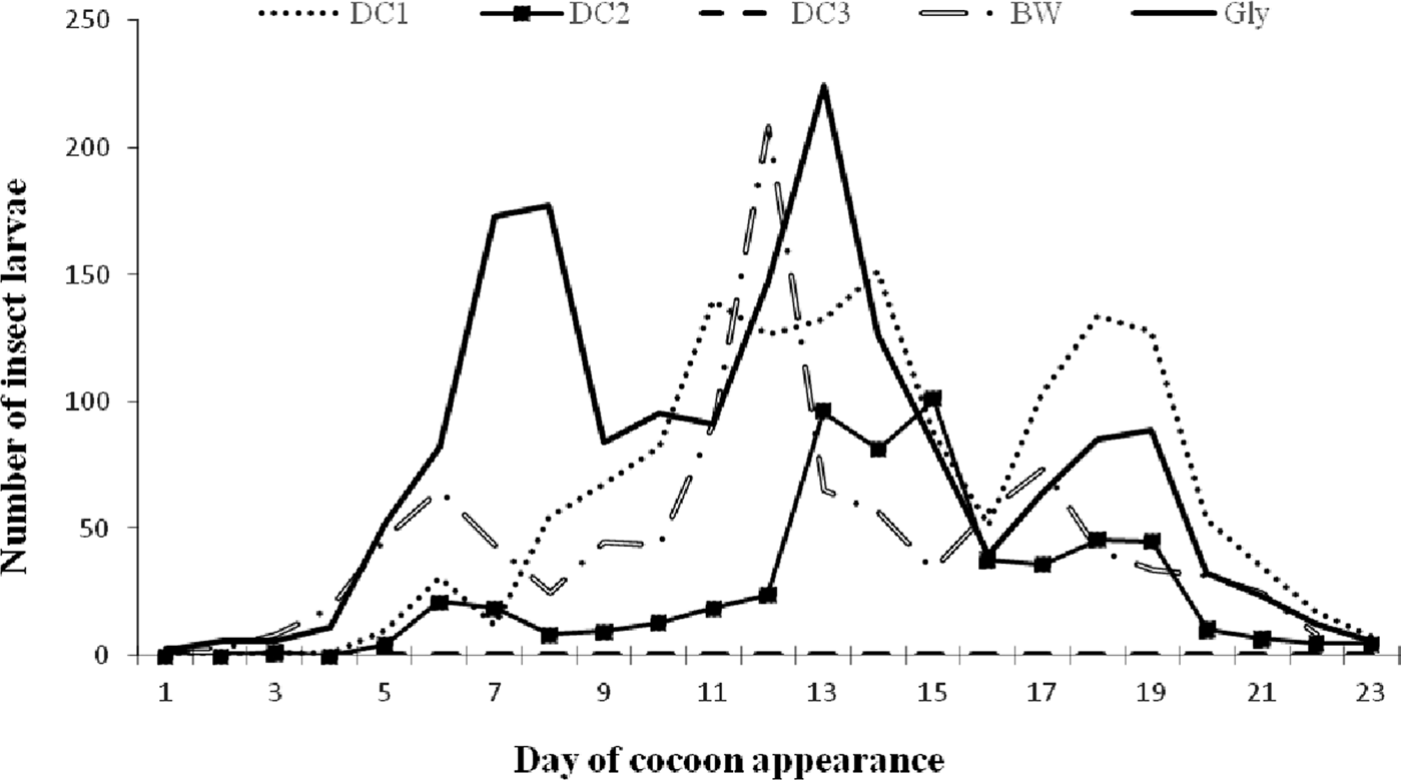
Variation curve of the number of *G. mellonella* larvae produced for all five diets as a function of time. BW, beeswax; Gly, standard glycerol-based artificial diet; DC1 to DC3, three varieties of adult dog croquettes.

Except for DC3, all diets (BW, Gly, DC1, and DC2) reached the stage of cocoon spinning with different average number of *G. mellonella* larvae produced according to the diet (Fig. 2). The maximum average number of cocoons (224.33 ± 62.26) was for the Gly diet at 13 d after young larvae were first observed in the breeding boxes. The lowest cocoon production was for diet DC2 during the first day of emergence and 13, 16, and 17, and then 20 to 23 d. During 13 to 15 and 17 to 19 d after observation of young larvae, the number of cocoons increased on this diet, and exceeding that for the diet BW, before decreasing. Diets BW, DC1, and Gly produced the highest number of larvae (Fig. 2).

### Cost of producing diets used to rear *G. mellonella* larvae

Table 2 shows the cost per kilogram of the different diets used to rear *G. mellonella* larvae. The Gly diet was the most expensive to produce at 6.30 US dollars (USD)/kg and could feed approximately 20 larvae for 1.5 wks. The cost of diet BW (5.11 USD/kg) was less than that of Gly and could be used to feed the same number of larvae for 3 wks. Diet DC3 was the least expensive of all diets (2.11 USD/kg) but did not favor any larval development. Diets DC1 and DC2 cost 2.72 and 2.88 USD/kg, respectively, and both these diets could feed 20 larvae for about 4 wks. In general, the diets incorporating dog croquettes were approximately half of the cost of the Gly and BW diets.

### Relationship between diet cost of production and weight of larvae

There was a low positive correlation (*r* = 0.44; non-significant, *P* = 0.4541) between the cost of production of the different diets and the mean weight of *G. mellonella* larvae at the cocoon spinning stage.

### Mortality caused by EPN to *G. mellonella* larvae fed on different diets

Significant differences in *G. mellonella* larval mortality were observed for diet (*P* < 0.001), exposure time (*P* < 0.01), and the interactions diet × nematode isolate (*P* < 0.001) and diet × nematode isolate × exposure time (*P* < 0.05) (Table 3).

**Table 3.** ANOVA for mortality at ambient temperature of *G. mellonella* larvae using factors of exposure time, nematode isolate, diet, and their interactions.

Source of variation	*F*	df	*P*
Isolate	0.34	4	0.851
Diet	9.91	2	0.001
Exposure time	5.42	3	0.002
Diet × isolate	5.49	8	0.001
Diet × exposure time	1.87	6	0.092
Isolate × exposure time	1.14	12	0.335
Diet × isolate × exposure time	1.62	24	0.048

Source of variation significant at P < 0.05.

**Figure 3:**
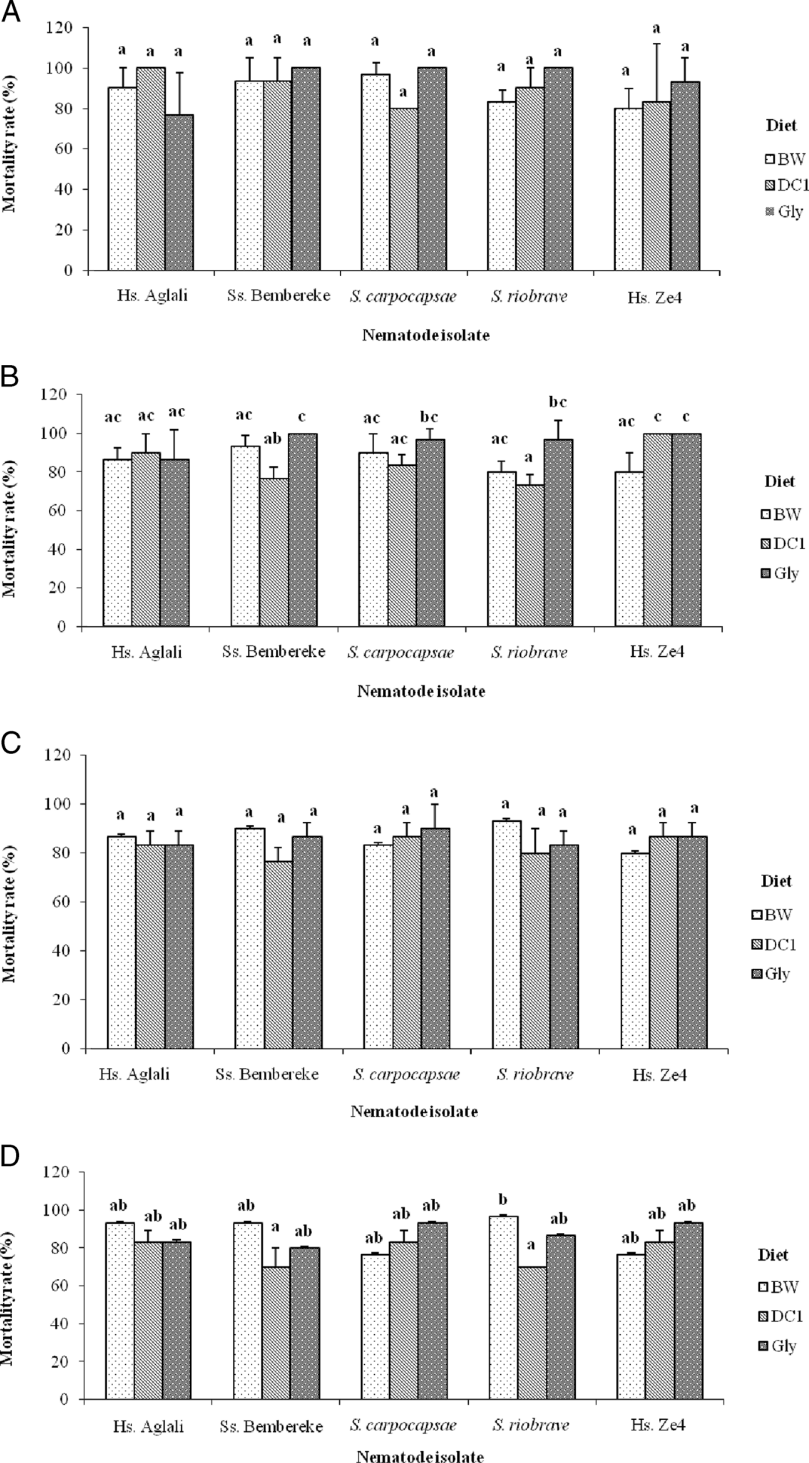
Mortality rate (mean ± standard deviation) of *G. mellonella* larvae based on diet, nematode isolate, and exposure time (T1-T4): (A) T1 (1 wk); (B) T2 (2 wks); (C) T3 (3 wks); and (D) T4 (4 wks). Mortality rates followed by the same letter do not significantly differ at *P* < 0.05. BW, beeswax; Gly, standard glycerol-based artificial diet; DC1, adult dog croquettes.

Diets Gly, BW, and DC1 affected the mortality caused by EPN isolates to *G. mellonella* larvae (Fig. 3A-D). At time periods T1 and T3 (Fig. 3A,C), there was no significant difference in mortality of larvae fed on the different diets and infected with different nematode isolates. However, at T2 (Fig. 3B) and T4 (Fig. 3D), larval mortality varied significantly (*P* < 0.05) when infected with nematode isolates. At T2, 100 ± 0.00% of larval mortality was observed with Bembereke (*Steinernema* sp.) and Ze4 (*H. sonorensis*) isolates fed on diet Gly; this mortality level was also observed on isolate Ze4 (*H. sonorensis*) fed on diet DC1. The lowest mortality rate (73.00 ± 5.77%) was for larvae fed on DC1 and inoculated with nematode isolate *S. riobrave*. The same nematode isolate caused the highest mortality rate (96.66 ± 5.77%) on diet BW at T4; and isolates Bembereke (*Steinernema* sp.) and *S. riobrave* caused the lowest mortality rates (70.00 ± 10.00% and 70.00 ± 0.00%, respectively) on diet BW.

### Population density of nematodes inside dissected *G. mellonella* larvae fed on each diet

Significant differences were observed for diet, nematode isolate, exposure time, and the interactions diet × nematode isolate, diet × exposure time, nematode isolate × exposure time, and diet × nematode isolate × exposure time (*P* < 0.001) (Table 4).

**Table 4.** ANOVA results for population density of nematodes in *G. mellonella* according to different factors (diets, nematode isolate, and exposure time) by dissection.

Source of variation	*F*	df	*P*
Diet	23.68	2	0.001
Nematode isolate	31.47	4	0.001
Exposure time	24.11	3	0.001
Diet × Nematode isolate	13.90	8	0.001
Diet × Exposure time	24.54	6	0.001
Nematode isolate × Exposure time	21.18	12	0.001
Diet × Nematode isolate × Exposure time	12.48	24	0.001

Source of variation significant at P < 0.05.

**Figure 4:**
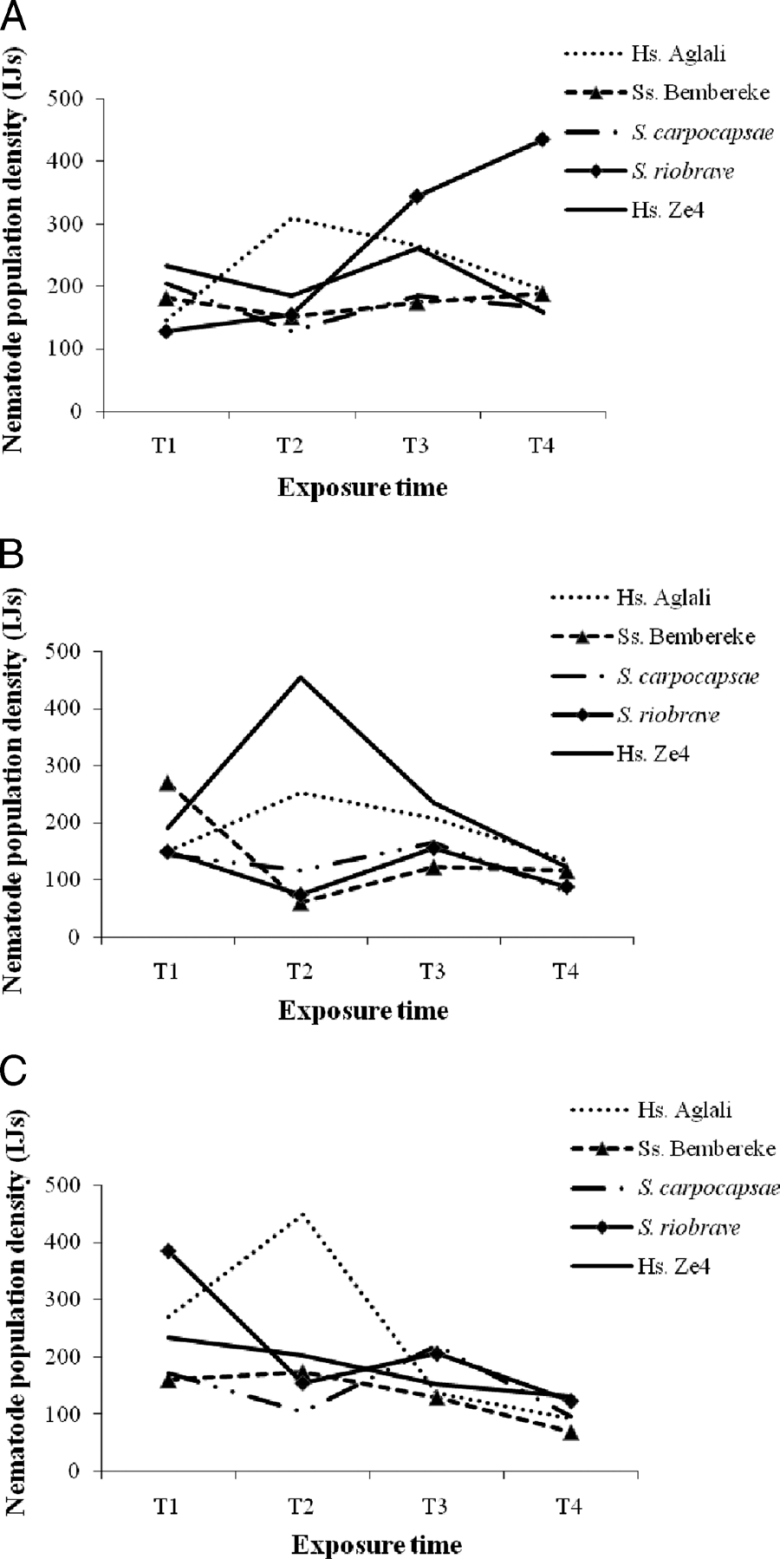
Nematode population density resulting from dissection of infected *G. mellonella* larvae fed on BW (beeswax), DC1 (adult dog croquettes), and Gly (standard glycerol-based artificial diet) over time (T1-T4). The nematode population densities presented are the means obtained in 50 μl of suspension/larvae: (A) BW; (B) DC1; and (C) Gly.

Regardless of diet and exposure time, IJs were observed inside the dissected infected *G. mellonella* larvae (Fig. 4A-C). The highest population densities of nematodes counted in 50 µl of suspension per infected larva were obtained at T2 with nematode isolate Aglali (*H. sonorensis*) on BW (309.11 ± 302.35 IJs/50 μl of suspension, 154,555 IJs/larva) (Fig. 4A) and Gly (447.83 ± 305.75 IJs/50  μl, 223,915 IJs/larva) (Fig. 4C) and isolate Ze4 (*H. sonorensis*) (452.96 ± 267.15 IJs/50  μl, 226,480 IJs/larva) on DC1 (Fig. 4B). At T4 on BW, the population densities of isolate *S. riobrave* increased and were the highest (*P* < 0.05), but those of isolates Aglali (*H. sonorensis*) and Ze4 (*H. sonorensis*) decreased. On DC1, however, *S. riobrave* gave the lowest population density at T1, T2, and T4, whereas at T3, the lowest population density of nematodes was for isolate Bembereke (*Steinernema* sp.). With diet Gly, isolates *S. carpocapsae* and Bembereke (*Steinernema* sp.) gave the lowest population densities of nematodes at T1 and T2 and during T2 to T4, respectively.

### Population density of nematodes emerging into White traps from infected larvae fed on different diets

Except for the interaction diet × exposure time (*F* = 1.08, df = 6, *P* = 0.3691), ANOVA showed that all factors and their interactions had significant effects: *P* < 0.05 for diet and *P* < 0.001 for nematode isolate, exposure time, diet × nematode isolate, nematode isolate × exposure time, and diet × nematode isolate × exposure time (Table 5).

**Table 5.** ANOVA results for population density of nematodes in *G. mellonella* according to different factors (diets, nematode isolate, and exposure time) by White traps.

Source of variation	*F*	df	*P*
Diet	4.33	2	0.013
Nematode isolate	64.61	4	0.001
Exposure time	145.79	3	0.001
Diet × Nematode isolate	6.82	8	0.001
Diet × Exposure time	1.08	6	0.369
Nematode isolate × Exposure time	11.23	12	0.001
Diet × Nematode isolate × Exposure time	4.78	24	0.001

Source of variation significant at P < 0.05.

**Figure 5:**
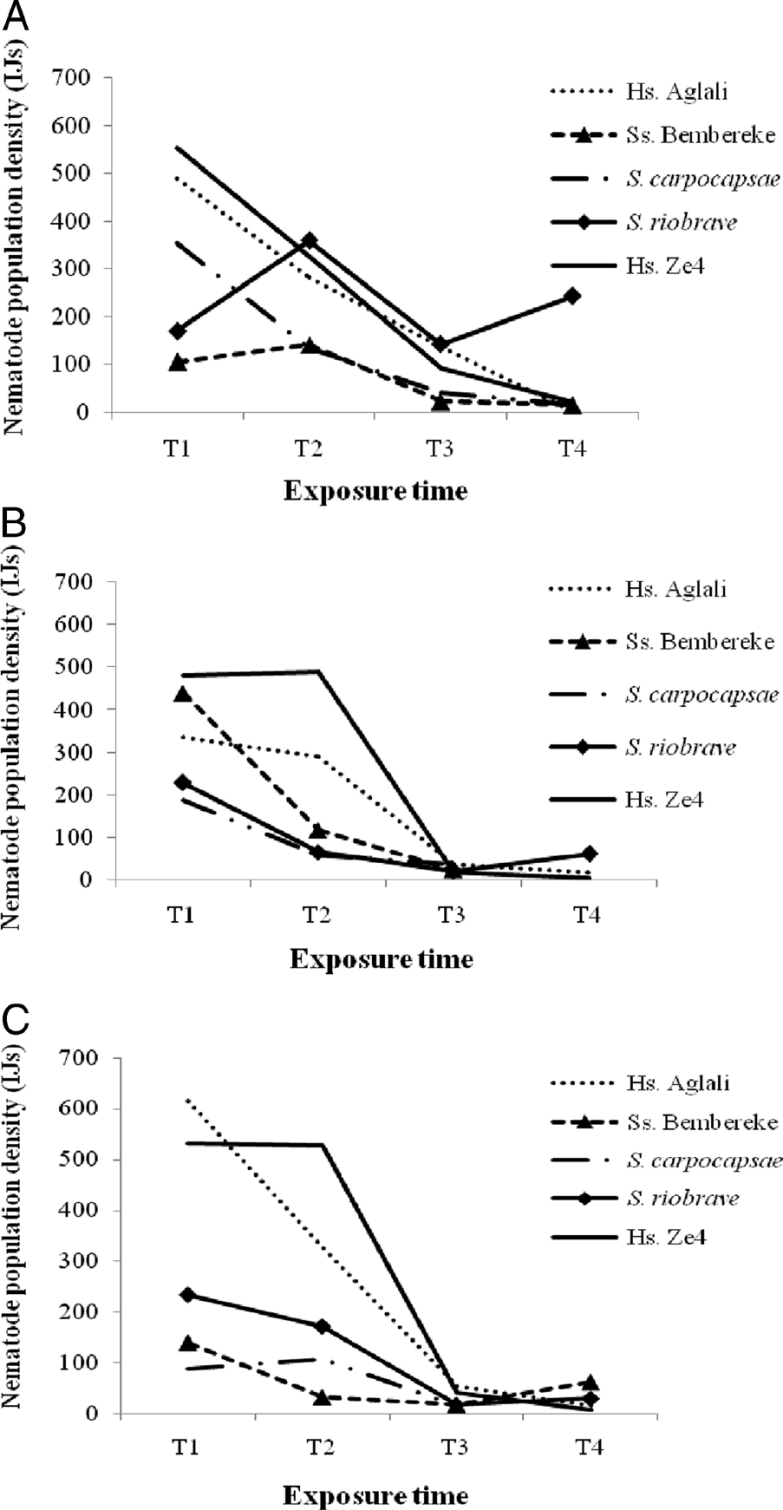
Nematode population density based on nematode isolate and exposure time (T1-T4), over 7 d after the first appearance of nematodes in White traps for the BW (beeswax), DC1 (adult dog croquettes), and Gly (standard glycerol-based artificial diet) diets. The nematode population densities presented are the means obtained in 50 μl of suspension/larvae: (A) BW; (B) DC1; and (C) Gly.

Population density of all five tested nematode isolates decreased with exposure time during T1 to T3, with the exception of isolates Bembereke (*Steinernema* sp.) and *S. riobrave* at T2 for diet BW (Fig. 5A-C). With up to 3 wks of exposure, all nematode isolates emerged from *G. mellonella* larvae fed on different diets.

With respect to larvae fed on diet BW, at T1 the nematode isolate Ze4 (*H. sonorensis*) had the highest nematode density (553.63 ± 311.97 IJs/50  μl, 276,815 IJs/larva) (Fig. 5A) and that of isolate Bembereke (*Steinernema* sp.) was the lowest (105.36 ± 84.22 IJs/50  μl, 52,680 IJs/larva). The population density of Aglali (*H. sonorensis*), Ze4 (*H. sonorensis*), and *S. carpocapsae* nematode isolates gradually decreased over time. All nematode isolates, with the exception of *S. riobrave*, had barely emerged at T4. The isolate Aglali (*H. sonorensis*) no longer emerged (0.00 ± 0.00 IJs/50  μl, 0 IJs/larva) at T4 from *G. mellonella* larvae. However, isolate *S. riobrave* continued to emerge and resulted in the highest nematode population density (242.95 ± 254.73 IJs/50  μl, 121,475 IJs/larva); its density increased during T1 to T2, but then decreased during T2 to T3 and again increased during T3 to T4.

The larvae fed on diet DC1, which had been infected and conserved until T4, did not favor the emergence of Bembereke (*Steinernema* sp.) and *S. carpocapsae* nematode isolates. On diet DC1, the highest population density of nematodes was for isolate Ze4 (*H. sonorensis*) (481.02 ± 2.97 IJs/50  μl, 240,510 IJs/larva) at T1 and the lowest density was for imported isolate *S. carpocapsae* (187.55 ± 0.00 IJs/50 μl, 93,775 IJs/larva) (Fig. 5B). The population density of Bembereke (*Steinernema* sp.), *S. carpocapsae*, and *S. riobrave* nematode isolates decreased progressively to T4 but *S. riobrave* increased from T3 to T4. However, this population density was generally stable during T1 to T2 for Ze4 and Aglali (*H. sonorensis*) isolates before progressively declining.

Nematode isolate Aglali (*H. sonorensis*) had the highest population density (615.18 ± 309.63 IJs/50 μl, 307,590 IJs/larva) at T1 on diet Gly among all diets. The lowest population density of nematodes at T1 was for isolate *S. carpocapsae* (87.83 ± 74.76 IJs/50 μl, 43,915 IJs/larva). At T4, isolate Bembereke (*Steinernema* sp.) had the highest population density (62.17 ± 33.60 IJs/50 μl, 31,085 IJs/larva) (Fig. 5C).

### Relationship between weight of larvae and population density of nematodes emerging

There was a significant positive correlation (*r* = 0.79, *P* = 0.0015) between the weight of *G. mellonella* larvae and the population density of nematodes from White traps.

### Relationship between costs of producing diets of *G. mellonella* and nematode population densities

**Figure 6:**
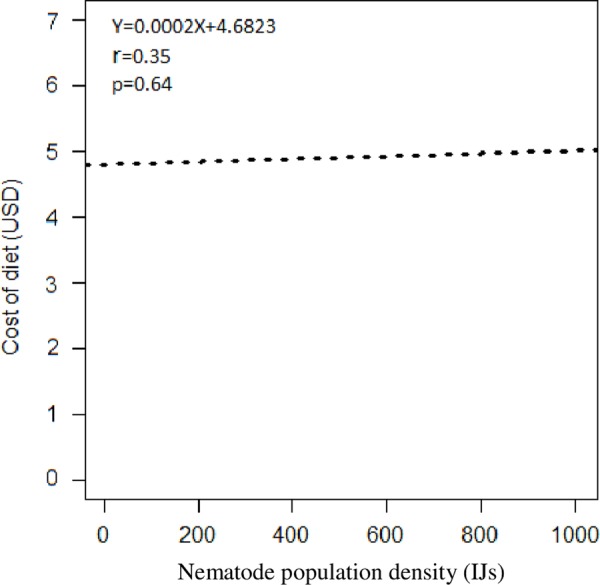
Relationship between costs of producing diets of *G. mellonella* and nematode population densities.

There was a positive (slope = 0.0002 ± 0.0005 > 0) but non-significant linear relationship between costs of producing diets of *G. mellonella* and nematode population density (*P* = 0.636) (Fig. 6).

## Discussion

Our investigations demonstrated the influence of diet (one natural and four artificial diets) on development and survival of *G. mellonella* larvae. Earlier studies had carried out mass-rearing of *G. mellonella* mostly on artificial diets to study various biological parameters such as duration of developmental life stages, fertility, and survival before choosing the tested materials as suitable diets (Chandel et al., 2003; Coskun et al., 2006; Birah et al., 2008; Kulkarni et al., 2012; Ellis et al., 2013). The best diet developed by Birah et al. (2008) for *G. mellonella* comprised wheat flour (130 g), wheat bran (130 g), milk powder (130 g), maize flour (97.5 g), yeast powder (97.5 g), beeswax (26 g), honey (195 ml), and glycerol (195 ml). Constituents similar to the above but in different proportions were also tested by Huang et al. (2010). In our study, Gly contained ingredients similar to those used by Birah et al. (2008) and Huang et al. (2010) and proved to be the most effective diet because it resulted in the highest mean number of *G. mellonella* larvae. Yeast has been frequently reported as a very important component in *G. mellonella* diets (Gross et al., 1996; Singh et al., 2014; Van Zyl and Malan, 2015). Gulati and Kaushik (2004) observed that factors such as relative humidity and diet influence *G. mellonella* development and metamorphosis. According to Cohen (2004), imperative to the rearing of high-quality hosts and EPNs is the selection of an artificial host diet that supports development of the entire life cycle of the host and outperforms other diets in terms of host yield production, weight accumulation, and developmental rate of hosts. Despite its effectiveness, the high cost of rearing *G. mellonella* larvae on the Gly diet remains a very important issue to resolve. This study showed that the diets differed in terms of larvae produced, with more expensive diets producing a higher weight of *G. mellonella* larvae. Moreover, diet Gly required more physical effort for preparation to rear *G. mellonella* larvae in the laboratory than any other diet evaluated.

The BW diet also resulted in good production of *G. mellonella* and can therefore be used as a substitute for Gly to rear the insects. It can feed the same number of larvae as Gly and for a longer period. Beeswax is easily obtained from beekeeping sites; however, in this study it did not allow a rapid production of *G. mellonella* larvae and therefore cannot be recommended when rapid production (i.e. within 3 wks) of insect larvae is needed for mass production of EPN. To overcome this problem, it is necessary to switch to another diet. Another problem with using beeswax as a diet is its limited availability only in the dry season on beekeeping sites. Coskun et al. (2006) found that the insects on a beeswax diet pupated earlier because of nutritional deficiency. According to NiemierKo and Wlodawer (1950), *G. mellonella* larvae can obtain a large amount of energy from beeswax, but it may not provide other nutrients in sufficient quantity, such as protein, for successful development. The diet DC3 did not favor emergence of *G. mellonella* larvae at all. Despite the presence of cereals in that diet, as also used in diets Gly and DC2, no *G. mellonella* larvae were observed. There are a number of dog croquette products on the market but they cannot all be used to rear *G. mellonella*. These findings agree with previous observations (Chandel et al., 2003; Birah et al., 2008; Kulkarni et al., 2012), who reported that detailed knowledge of an insect’s biology is necessary to evaluate artificial diet modifications. In some cases, brands of dog food have insecticides added to the formulation as a protective treatment against fleas (Wallinga and Greer, 2000), thus making them useless for insect rearing. The diet DC3 may contain such products and this could explain the lack of development of *G. mellonella* larvae.

For a small-scale laboratory production intended, for example, for conservation of isolates of EPN, diet DC1 could be considered because it produced a greater number of larvae and more quickly than diet DC2. However, there are some difficulties associated with the use of dog croquettes for *G. mellonella* larvae rearing because of the scarcity of certain brands of croquettes on the market for extended periods. Dog croquettes DC1 and DC2 favored the development of *G. mellonella* larvae and this could be explained by their inclusion of beef and chicken (or other poultry) which were absent from DC3.Thus, the type of diet and its composition influenced development of *G. mellonella*.

Diet DC1 is economically profitable for mass-rearing *G. mellonella* but larval development was less rapid compared with Gly. The choice of diet to adopt in this context seems to be a function of available diets and the materials to prepare them. We recommend Gly for *G. mellonella* larvae mass-rearing in the laboratory when EPN are to be applied in infected insect larvae for biological control trials in the field. However, because of the high cost of this diet, BW and DC1 can be used instead of Gly if a program of larvae application in the field has previously been well established in advance. To maintain EPN isolates in the laboratory, DC1 can be used to rear *G. mellonella* because this diet has a low cost and the weight of larvae does not significantly differ from those fed on BW and Gly. When considering the independence of sweet potato producers in the context of biological control, beeswax will more useful than the other diets because some producers have beekeeping sites or friends raising bees from which they can easily obtain beeswax (the natural diet of the insect). For those growers for whom acquisition of beeswax is difficult, diet DC1 is preferable and DC2 is an alternative.

Concerning the mortality of larvae, it is possible that the diet fed to *G. mellonella* affects the efficiency in killing the host by the EPN multiplied within them. According to Finke (2002), *G. mellonella* larvae contain a high percentage of fat, and certain lipid components that have been shown to be conducive in increasing the developmental rate and yield of *Heterorhabditis* nematodes. Shin et al. (2001) analyzed the lipid content of *G. mellonella* exposed to different concentrations of cadmium chloride independently contaminating an artificial diet at different concentrations. Their results showed that lipid levels decreased in the cadmium-contaminated groups compared with the control group. Thus, the lipid composition and perhaps other compounds in the body of *G. mellonella* larvae vary with the diet consumed. According to Andaló et al. (2011), lipids represent the main source of energy for EPN; in the IJ stage, the level of such reserves can be influenced by storage, and this may affect their infectivity, while IJ is the only infective stage of nematodes. The diet used to feed *G. mellonella* affected population density of nematodes and this also varied with time. Therefore, the diet on which the host feeds also influences the efficacy of the parasite. Sajjan (2016) evaluated the yield of *H. indica* from *G. mellonella* larvae reared on five diets and found that the population density of nematodes for these diets followed different patterns. This could explain the highest densities of nematodes obtained from *G. mellonella* larvae at T1 into White traps with isolate Ze4 on BW and DC1, and Aglali on Gly, which both belong to the genus *Heterorhabditis*. The *G. mellonella* larvae fed on the BW diet continued to produce nematodes of isolate *S. riobrave* beyond T4.

The findings of this study suggest that diet is one of the most important factors influencing development of *G. mellonella*. Also, it should be noted that the choice of diet, particularly with regard to dog croquettes, for rearing the insects as well as diet composition are important factors in planning breeding of *G. mellonella*. We conclude that the reproductive potential of EPN and their pathogenicity vary over time depending on the diet fed to *G. mellonella* larvae.
